# Association between anti-mullerian hormone and metabolic syndrome: insights from a prospective community-based study

**DOI:** 10.1186/s12902-024-01627-z

**Published:** 2024-06-27

**Authors:** Mina Amiri, Maryam Mousavi, Mahsa Noroozzadeh, Maryam Farahmand, Fereidoun Azizi, Fahimeh Ramezani Tehrani

**Affiliations:** 1 Reproductive Endocrinology Research Center, Research Institute for Endocrine Sciences, Shahid Beheshti University of Medical Sciences, 23 Arabi, Yaman Street, Velenjak Tehran, Iran P.O. Box, 19395-4763; 2 The Foundation for Research and Education Excellence, Vestavia Hills, AL USA; 3 Endocrine Research Center, Research Institute for Endocrine Sciences, Shahid Beheshti University of Medical Sciences, Tehran, Iran

**Keywords:** Metabolic syndrome (MetS), Anti-mullerian hormone (AMH), Community-based study

## Abstract

**Background:**

Limited studies have investigated the relationship between Anti-Müllerian hormone (AMH) and metabolic syndrome (MetS), yielding inconclusive results. This study aimed to examine the relationship between AMH levels and MetS and its components in women from a general population.

**Methods:**

This prospective study recruited 769 women. Generalized Estimating Equation (GEE) models analyzed longitudinal trends of MetS components. Cox proportional hazard models evaluated effect of age-specific AMH tertiles on MetS occurrence, adjusting for confounders.

**Results:**

The GEE analysis indicated that women in the third tertile exhibited higher mean FPG compared to those in the first tertile of age-specific AMH (3 mg/dL; 95% CI: 0.40, 5.60; *P* = 0.024); however, this association became non-significant after adjustment. Notably, the second tertile showed a significant decrease in FPG mean changes over time (-0.69 mg/dL; 95% CI: -1.31, -0.07; P _Interaction_ = 0.030). Women in the second and third tertiles of age-specific AMH demonstrated lower mean HDL-C compared to the first tertile (-2.96 mg/dL; 95% CI: -4.67, -1.26; *P* < 0.001 and -2.63 mg/dL; 95% CI: -4.31, -0.96; *P* = 0.002, respectively). The association between HDL-C changes and the second tertile remained significant after adjustment (-1.91 mg/dL; 95% CI: -3.68, -0.14; *P* = 0.034). No significant associations were observed between age-specific AMH tertiles and TG and SBP/DBP. Cox models revealed no significant differences in the hazard ratio of MetS between AMH tertiles after adjusting for confounders.

**Conclusion:**

Despite minor variations in MetS components, AMH levels did not affect MetS risk in women from a general population.

## Introduction

Metabolic syndrome (MetS) is a cluster of metabolic abnormalities including central obesity, hypertension (HTN), hyperglycemia, and dyslipidemia, which frequently used as a proxy for predicting the risk of these potentially life-threating diseases, such as cardiovascular disease (CVD) and type 2 diabetes (T2D) [1]. The prevalence of MetS is increasing worldwide in both men and women [2]. However, several studies have demonstrated that postmenopausal women, particularly those with premature ovarian insufficiency (POI), are at an increased risk developing MetS than premenopausal women [1, 3, 4]. The higher prevalence of MetS in postmenopausal women with undetectable or diminished ovarian reserve raises an intriguing hypothesis that there may be shared underlying factors contributing to MetS and ovarian reserve [5].

Anti-Müllerian hormone (AMH) is a type of dimeric glycoprotein which is typically produced by the granulosa cells of preantral and small antral follicles throughout the reproductive lifespan and serves as the most reliable indicator of ovarian reserve [6]. There is growing evidence that beyond reproductive implications, AMH may be involved in the pathogenesis of metabolic disorders [7]. As revealed by our earlier study, incorporating serum AMH concentrations into the Framingham Risk Score (FRS) and Pooled Cohort Equations (PCE) risk prediction tools improves the accuracy of predicting CVD risk. This highlights the significant potential of using this biomarker as a valuable tool to predict cardiometabolic risk in women [8].

There are limited studies directly investigating the relationship between AMH levels and MetS and its components in the general population; most research has focused on women with polycystic ovary syndrome (PCOS) [5, 7, 9–19]. It is well-documented that PCOS presents with higher serum concentrations of AMH and an increased risk of obesity and other metabolic disorders [9, 10, 17]. The inclusion of PCOS-related evidence in a general population study may lead to erroneous conclusions or inappropriate comparisons.

To address this gap in knowledge, we conducted a community-based prospective study with long-term follow-up to investigate the association between age-specific AMH tertiles and developing MetS in a general population of women. We also aimed to compare the trend of components of MetS overtime in women with different levels of age-specific AMH. Finding from this study will provide novel insights into the role of AMH in the pathogenesis of metabolic disorders and further highlight the importance of early prevention and management of metabolic disturbances in women with PCOS.

## Materials and methods

### Study design and participants

This study was conducted using data collected from participants of the Tehran Lipid and Glucose Study (TLGS), a long-term prospective cohort established in 1998. The TLGS assesses various risk factors for non-communicable diseases, demographic variables, and reproductive histories through face-to-face interviews every three years in six follow-up visits. For this specific study, women aged 20 years or older who participated in the baseline and at least one follow-up visit and had regular and predictable menstrual cycles at the initiation of the study were included. Among the 1,015 women who participated in TLGS and had age-specific AMH records at baseline, 246 were excluded due to pre-existing MetS at baseline (n = 241), or having less than two follow-ups with information on MetS (n = 5). Therefore, the study analyzed 769 eligible women. Out of these, 429 women developed MetS during the study, while 340 women did not experience MetS by the end of the observation period.

### Measurements

All study participants were interviewed to obtain medical, obstetrics, and family histories using pretested questionnaires. Clinical and anthropometric measurements were assessed by trained examiners at each follow-up.

The study conducted various biochemical measurements at baseline and during follow-up visits. Serum AMH levels were only tested at baseline, while all other measurements were taken at each visit. All blood samples were collected in the morning after a 12-h overnight fast and analyzed on the day of collection at the TLGS research laboratory. All sera were stored at –80 °C until the time of testing. The AMH concentration was measured using the two-site enzyme immunoassay (EIA) method with the Gen II kit from Beckman Coulter. Fasting plasma glucose (FPG), triglycerides (TG), and high-density lipoprotein cholesterol (HDL-C) were also measured using enzymatic colorimetric methods with related kits from Pars Azmon Inc., Iran. HDL-C was measured after apolipoprotein B-containing lipoproteins were removed with phosphotungistic acid. Assay performance was monitored using lipid control serum, Precinorm, and Precipath, and lipid standard was used to calibrate the Selectra 2 auto-analyzer for all laboratory analyses. The coefficients of variation (CVs) for AMH were calculated separately for intra-assay and inter-assay measurements, resulting in values of 1.9% and 2.0%, respectively. Similarly, the CVs for glucose, total and HDL-C, and TG were also determined for both intra-assay and inter-assay measurements, with glucose having an intra- and inter-assay CV of 2.2%, while HDL-C had intra-assay CVs of 0.5% and 2%, respectively. Lastly, TG had intra-assay and inter-assay CVs of 0.6% and 1.6%, respectively.

### Term definition

Smoking status was classified into two categories, including ever smokers (current users and those who used to smoke in the past) and never smokers. For evaluating physical activity, a modified activity questionnaire (MAQ) was used, which is evaluated and validated in the Iranian population. According to the questionnaire, physical activity has been specified as low (MET < 600 min/wk), moderate (MET 600—1499 min/wk), and high (MET ≥ 1500 min/wk) levels [20]. A positive family history of diabetes was considered as having previously diagnosed diabetes in relatives.

### Exposure

The study has categorized female participants into three groups based on their age-specific AMH levels. These groups correspond to tertiles of the population distribution, which divide subjects into three equal-sized groups based on a specific variable (in this case, age-specific AMH levels).

### Outcome

The criteria for diagnosing MetS involve the presence of at least three of five key risk factors, which have been established based on the Joint Interim Statement [1]. These risk factors include abdominal obesity, as determined by a waist circumference of at least 95 cm, with specific cutoffs for Iranians [21], FPG levels equal to or greater than 100 mg/dL (or drug treatment), TG levels equal to or greater than 150 mg/dL (or drug treatment), low levels of HDL-C (less than 50 mg/dL in women, or drug treatment), and elevated BP, defined as systolic blood pressure (SBP) equal to or greater than 130 mm Hg, diastolic blood pressure (DBP) equal to or greater than 85 mm Hg, or antihypertensive drug treatment.

### Statistical analysis

We described and compared the baseline characteristics of the participants in three tertiles of AMH. To determine normality assumptions in continuous variables, we used the Kolmogorov–Smirnov test. We used mean [standard deviation (SD)] and ANOVA tests for variables with normal distribution, and median [interquartile range (IQR)] and Kruskal–Wallis tests for those without normality assumptions. For categorical variables, we reported frequencies (%) and used the Chi-squared test or Fisher exact test to compare these variables in the three groups.

The age-related decline in AMH levels is well-established in the literature [22, 23]; this age-dependent variation in AMH concentrations precludes the use of a single reference value for the entire study population. Furthermore, several studies have documented a non-linear pattern of AMH decrease with advancing age in women [24–26]. For the purpose of the present study the age-specific AMH percentiles have been calculated using the normal-based methodology introduced by Altman and Chitty [27], and Royston and Wright [28]. For this calculation, the Fractional polynomial (FP) regression models were fitted separately to estimate the mean and SD of the log AMH values as functions of age. An exponential – normal (EN) 3-parameter model provided the most fitted results since 9.8% of the observations lie above the 90th percentile and 9.1% below the 10th percentile [26]. For the purpose of the present study, we classified our participants to three group according to their age-specific AMH tertiles, and compared their baseline characteristics.

To evaluate the Hazard Ratio (HR) of occurrence of MetS in various age-specific AMH tertiles, we used the Cox proportional hazard model. The proportional hazard assumption of Cox model were tested for age specific AMH tertiles and other variables in final model. We also investigated the secular longitudinal trends of five MetS components [WC (cm), FPG (mg/dl), HDL-C (mg/dl), SBP (mmHg), and DBP (mmHg)] in our follow-ups and the effects of factors on these trends using Generalized Estimating Equation models (GEE). The GEE modeling accounts for correlations within subjects through a working correlation matrix and enables investigators to estimate the effect size accurately, even in the presence of incomplete data which is common in cohort studies due to missing variables in some repeated measures. We reported the results of both unadjusted and adjusted models for Cox and GEE models by considering several potential confounders, including age, educational level, physical activity, smoking status, and family history of diabetes.

All statistical analyses were performed in STATA (version 12; STATA Inc., College Station, TX, USA), and p-values less than 0.05 were considered statistically significant.

## Results

A total of 769 eligible women were followed up for a median of 16 years (IQR: 9–19). Table 1 presents the baseline characteristics of the study participants with and without MetS. Women who developed MetS had a higher median (IQR) age compared to those without MetS [36 (32–41) vs. 32 (28–37 years); *p* < 0.001]. Furthermore, women with MetS had a higher BMI compared to those without this syndrome [median (IQR) 27.23 (24.43–29.42) vs. 24.22 (22.01–26.83) kg/m^2^; *p *< 0.001]. Additionally, women who experienced MetS had a higher percentage of family history of diabetes compared to those without this syndrome (34% vs. 22.6%; *p* = 0.01). During follow-ups, women with MetS exhibited significantly higher levels of WC [median (IQR) 85 (79–90) vs. 79 (73–85) cm; *p* < 0.001], FPG [median (IQR) 87(82–92) vs. 85 (80–90) mg/ml p < 0.001], TG [median (IQR) 115 (88–147) vs. 85.5 (68–105) mg/dl; *p* < 0.001], SBP [median (IQR) 110 (102–118) vs. 105 (100–111) mmHg; *p* < 0.001], and DBP [median (IQR) 75 (70–80) vs. 71 (66.75–70) mmHg; *p* < 0.001] than healthy women. Conversely, they exhibited lower levels of HDL-C [median (IQR) 42 (35–49) vs. 46 (39.75–53) mg/dl; *p *< 0.001].

**Table 1:** Baseline characteristics of the study participants according to the occurrence of metabolic syndrome during follow-up

**Covariates and metabolic syndrome components**	**Metabolic syndrome status over follow-ups**		***P*-value**
	**No (*n*=340)**	**Yes (***n***=429)**	
**Age (Years), Median (IQR)**	32 (28-37)	36 (32-41)	< 0.001^*^
**BMI (kg/m**^**2**^**), Median (IQR)**	24.22 (22.01-26.83)	27.23 (24.43-29.42)	< 0.001^*^
**Age specific AMH, Median (IQR)**	0.47 (0.23-0.72)	0.50 (0.24-0.76)	0.250
**Educational level (years), N (%)**			
<6	3 (0.9)	5 (1.2)	0.133
6-12	286 (84.1)	380 (88.6)	
>12	51 (15)	44 (10.3)	
**Smoking, N (%)**			
Never	326 (95.9)	409 (95.3)	0.715
Ever	14 (4.1)	20 (4.7)	
**Physical activity, N (%)**			
Low	246 (72.6)	292 (68.4)	0.209
Moderate to high	93 (27.4)	135 (31.6)	
**Diabetes status, N (%)**			
No	339 (99.7)	442 (98.4)	0.069
Yes	1 (0.3)	7 (1.6)	
**Family history of diabetes, N (%)**			
No	263 (77.4)	283 (66)	< 0.001^*^
Yes	77 (22.6)	146 (34)	
**WC (cm)**	79 (73-85)	85 (79-90)	< 0.001^*^
**FPG (mg/dl)**	85 (80-90)	87 (82-92)	< 0.001^*^
**HDL-C (mg/dl)**	46 (39.75-53)	42 (35-49)	< 0.001^*^
**TG (mg/dl) **	85.5 (68-105)	115 (88-147)	< 0.001^*^
**SBP (mmHg)**	105 (100-111)	110 (102-118)	< 0.001^*^
**DBP (mmHg)**	71 (66.75-77)	75 (70-80)	< 0.001^*^

*Abbreviations*: *WC* Waist circumference, *FPG* Fasting plasma glucose, *TG* Triglyceride, *HDL-C* High-density lipoprotein cholesterol, *SBP* Systolic blood pressure, *DBP* Diastolic blood pressure

*Significant values

**Fig. 1:**
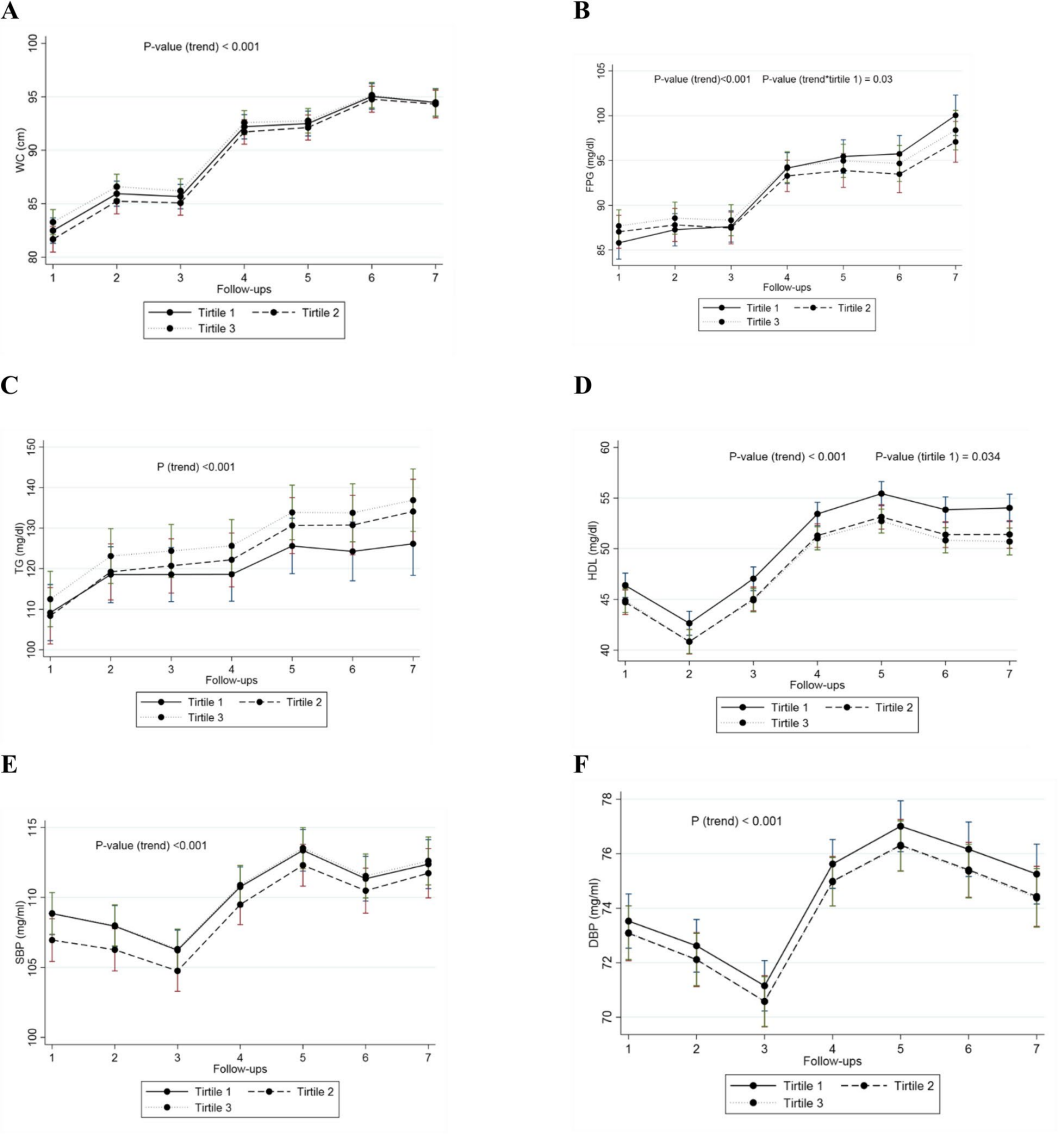
**A**-**F** Trends in metabolic syndrome (MetS) components over time based on age-specific anti-mullerian hormone (AMH) tertiles. **A** Waist circumference (WC) trends over time based on age-specific AMH tertiles. **B** Fasting plasma glucose trends over time based on age-specific AMH tertiles. **C** Triglyceride trends over time based on age-specific AMH tertiles. **D** High-density lipoprotein cholesterol trends over time based on age-specific AMH tertiles. **E** Systolic blood pressure trends over time based on age-specific AMH tertiles. **F** Diastolic blood pressure trends over time based on age-specific AMH tertiles

Table 2 presents the results of GEE models that estimated the effect of age-specific AMH tertiles on MetS components during follow-ups. The mean WC in women in the second and third tertiles of age-specific AMH did not significantly differ from those in the first tertile. Our study found that women in the third tertile of age-specific AMH had slightly higher mean FPG than those in the first tertile of age-specific AMH (3 mg/dl; 95% CI: 0.40, 5.60; P = 0.024); however, adjusting for confounders eliminate this association. For each follow-up visit, women who were in second and third age-specific AMH tertiles had lower mean change in FPG compared to those who were in the first teritle (-0.76 mg/dl; 95% CI: -1.36, -0.16; P _Interaction_ = 0.013) and (-0.62 mg/dl; 95% CI: -1.21, -0.03; P _Interaction_ = 0.040), respectively. However, this interaction effect remained statistically significant for the time and the second tertile of age-specific AMH (-0.69 mg/dl; 95% CI: -1.31, -0.07; P _Interaction_ = 0.030) after adjusting for confounders. The mean TG over time did not significantly differ in women in the second and third tertiles of age-specific AMH compared to those in the first tertile, and this finding remained non-significant even after adjusting for confounders. Conversely, women in the second and third tertiles of age-specific AMH had lower means of serum levels of HDL-C than those in the first tertile (-2.96 mg/dL; 95% CI: -4.67, -1.26; P < 0.001 and -2.63 mg/dl; 95% CI: -4.31, -0.96; P = 0.002, respectively). However, after adjusting for confounders, this association remained significant only for the second tertile of age-specific AMH (-1.91 mg/dl; 95% CI: -3.68, -0.14; P = 0.034). The interaction between time and age-specific AMH tertile on the HDL-C was not significant. While both SBP and DBP exhibited upward trends, irrespective of age-specific AMH levels, there were no notable variances in the average SBP and DBP over time between women categorized in the second and third tertiles of age specific AMH compared to those in the first tertile. Furthermore, the interaction effect between time and age-specific AMH tertile on the mean alterations in BP was not statistically significant. Figures 1 A-F depict the temporal patterns of MetS components across the various tertiles of age-specific AMH.

**Table 2:** The results of GEE models to estimate the effect of age-specific AMH tertiles on metabolic syndrome components over time

**Variables**		Model 1^*^	***P*-value**	Model 2^**^	***P*-value**
		**Coefficient (95% CI)**		**Coefficient (95% CI)**	
**WC (cm)**	T_1_(ref.)	-	-	-	-
	T_2_	-1.16 (-2.93, 0.62)	0.201	-0.93 (-2.68, 0.82)	0.297
	T_3_	0.67 (-1.08, 2.42)	0.450	0.86 (-0.87, 2.59)	0.329
	Time	2.28 (2.10, 2.47)	< 0.001^***^	2.30 (2.12, 2.48)	< 0.001^***^
	Time × T_2_	0.11 (-0.15, 0.37)	0.419	0.11 (-0.15, 0.37)	0.407
	Time × T_3_	-0.12 (-0.38, 0.14)	0.349	-0.13 (-0.39, 0.12)	0.310
**FPG (mg/dl)**	T_1_(ref.)	-		-	-
	T_2_	2.57 (-0.07, 5.21)	0.057	1.90 (-0.79, 4.60)	0.166
	T_3_	3 (0.40, 5.60)	0.024^***^	2.39 (-0.25, 5.04)	0.076
	Time	2.43 (2, 2.86)	< 0.001^***^	2.35 (1.91, 2.78)	< 0.001^***^
	Time × T_2_	-0.76 (-1.36, -0.16)	0.013^***^	-0.69 (-1.31, -0.07)	0.030^***^
	Time × T_3_	-0.62 (-1.21, -0.03)	0.040^***^	-0.58 (-1.19, 0.03)	0.063
**TG (mg/dl)**	T_1_(ref.)	-		-	-
	T_2_	-2 (-12.25, 8.25)	0.702	-2.20 (-12.50, 8.08)	0.674
	T_3_	1.98 (-8.15, 12.11)	0.702	2.21 (-7.96, 12.38)	0.670
	Time	2.39 (1.06, 3.72)	< 0.001^***^	2.39 (1.06, 3.72)	< 0.001^***^
	Time × T_2_	1.39 (-0.50, 3.29)	0.150	1.47 (-0.43, 3.36)	0.130
	Time × T_3_	1.23 (-0.63, 3.09)	0.195	1.23 (-0.63, 3.10)	0.195
**HDL-C (mg/dl)**	T_1_ (ref.)	-		-	-
	T_2_	-2.96 (-4.67, -1.26)	0.001^***^	-1.91 (-3.68, -0.14)	0.034^***^
	T_3_	-2.63 (-4.31, -0.96)	0.002^***^	-1.66 (-3.40, 0.07)	0.060
	Time	1.84 (1.61, 2.06)	< 0.001^***^	1.77 (1.54, 1.99)	< 0.001^***^
	Time × T_2_	-0.05 (-0.37, 0.26)	0.735	-0.14 (-0.46, 0.17)	0.361
	Time × T_3_	-0.19 (-0.51, 0.12)	0.223	-0.28 (-0.59, 0.03)	0.078
**SBP (mmHg)**	T_1_ (ref.)	-	-	-	
	T_2_	-2.21 (-4.54, 0.13)	0.064	-1.95 (-4.20, 0.30)	0.089
	T_3_	0.29 (-2.01, 2.59)	0.802	0.01 (-2.20, 2.22)	0.993
	Time	0.88 (0.56, 1.20)	< 0.001^***^	0.86 (0.55, 1.18)	< 0.001^***^
	Time × T_1_	0.20 (-0.25, 0.65)	0.377	0.19 (-0.26, 0.64)	0.405
	Time × T_3_	0.002 (-0.44, 0.45)	0.994	0.02 (-0.42, 0.46)	0.918
**DBP (mmHg)**	T_1_(ref.)	-		-	-
	T_2_	-0.31 (-1.89, 1.28)	0.705	-0.31 (-1.84, 1.22)	0.690
	T_3_	-0.20 (-1.76, 1.37)	0.805	-0.35 (-1.86, 1.16)	0.649
	Time	0.67 (0.46, 0.88)	< 0.001^***^	0.65 (0.44, 0.85)	< 0.001^***^
	Time × T_2_	-0.10 (-0.39, 0.19)	0.496	-0.07 (-0.36, 0.22)	0.628
	Time × T_3_	-0.09 (-0.38, 0.19)	0.522	-0.08 (-0.37, 0.20)	0.573

*Abbreviations: AMH* Anti-mullerian hormone, *T* Tertile of age-specific *AMH*, *T1* The first tertile of age-specific *AMH*, *T2* The second tertile of age-specific *AMH*, *T3* The third tertile of age specific *AMH*, *ref* Reference level, *WC* Waist circumference, *FPG* Fasting plasma glucose, *TG* Triglyceride, *HDL-C* High-density lipoprotein cholesterol, *SBP* Systolic blood pressure, *DBP* Diastolic blood pressure

^*^Unadjusted model

^**^Adjusted models for baseline variables: age, BMI (except for WC), physical activity, educational level, smoking status, family history of diabetes status

^***^Significant values

In the unadjusted Cox models, HR for MetS did not display any significant differences among women in the second and third tertiles of age-specific AMH relative to those in the first tertile. Following adjustments made for potential confounding factors, the findings indicate that there was no discernible disparity in the HR for MetS between women grouped within these tertiles of age-specific AMH (Table 3). The proportionality hazard assumption was valid for AMH (P = 0.031); all the other adjusted variables in our Cox model have not violated from this assumption.

**Table 3:** Cox models hazard ratios (HRs) of the occurrence of metabolic syndrome based on the different age-specific AMH tertiles

**Variables**		Model 1*		Model 2**	
		**HR (95% CI)**	***P*-value**	**HR (95% CI)**	***P*-value**
**Age-specific AMH, tertiles**					
	**T**_**1**_** (Ref.)**	-	-	-	-
	**T**_**2**_	0.86 (0.92–1.48)	0.184	0.90 (0.91–1.14)	0.392
	**T**_**3**_	1.26 (0.99–1.59)	0.060	1.09 (0.86–1.38)	0.433

*Abbreviations: AMH* Anti-mullerian hormone, *T* Tertile of age-specific AMH, *T1* The first tertile of age-specific AMH, *T2* The second tertile of age-specific AMH, *T3* The third tertile of age specific AMH, *ref* Reference level, *HR* Hazard ratio

^*^Unadjusted model

^**^Adjusted model for age, BMI, physical activity, educational level, smoking status, family history of diabetes status

## Discussion

Our study aimed to elucidate the relationship between MetS and AMH using data from a community-based prospective study with ~ 20 years follow-up. The results of our analysis revealed intriguing associations between individual components of MetS and age-specific AMH levels, highlighting the intricate interplay between hormonal imbalances and metabolic dysfunction. Notably, we did not find any significant association between the occurrence of MetS as an event and levels of age-specific AMH, even after adjusting for several potential confounders.

Numerous studies have examined the relationship between metabolic parameters and AMH in women, particularly those with PCOS. However, these studies have yielded conflicting findings. For instance, one cross-sectional study of reproductive-age women with PCOS found a positive association between serum AMH levels and HOMA IR levels [10]. Another cross-sectional analysis of 252 women aged 18–46 with PCOS revealed that AMH levels correlated positively with HDL-C cholesterol and negatively with fasting glucose, insulin resistance, BMI, SBP, and DBP. The study suggests that low AMH levels in young women with PCOS may predict a higher risk of MetS [7]. Conversely, a cross‑sectional study conducted in India on women diagnosed with PCOS aged 20–40 found no correlation between serum AMH levels and any component of MetS [13].

Limited research has explored the association between AMH and metabolic disturbances in the general population, yielding inconsistent findings [5, 15, 16, 29, 30]. For instance, a cross-sectional study conducted with 136 participants found that ovarian reserve function was significantly lower in individuals with MetS, particularly among women aged 20–29 [31]. Another cross-sectional analysis revealed lower AMH levels in women with T2D compared to those without T2D, specifically before age of 35 [15]. Similarly, a separate cross-sectional study involving non- PCOS women demonstrated an independent inverse relationship between insulin, fasting glucose, HOMA-IR, and AMH [16]. Additionally, a study examining women with diminished ovarian reserve and those with normal ovarian reserve discovered positive associations between low AMH levels and metabolic parameters such as HOMA-IR, CRP, TG, and LDL-C levels, while observing a negative correlation with HDL-C [5]. Conversely, a cross-sectional study comprising 291 women late reproductive age did not identify a significant correlation between MetS risk components and serum AMH levels [32]. However, prospective studies yielded inconsistent results. For example, our previous study indicated that women with lower ovarian reserve did not demonstrate distinct trends in adiposity and glucose metabolism parameters over their reproductive life span [29]. Furthermore, a prospective cohort study involving 3,293 women between the ages of 20 and 59 did not provide clear evidence of differences in AMH trajectories between women who developed T2D and those who did not [30]. The majority of available studies did not adjust their findings for essential confounders, which may be the reason for the controversy among studies. Additionally, a couple of studies have assessed the relationship between AMH and metabolic parameters in both PCOS and non-PCOS, separately. For instance, cross sectional data from eight US-based academic centers demonstrated intriguing associations among women with PCOS. It revealed that AMH displayed an inverse correlation with important indicators such as BMI, WC, fasting insulin, HOMA-IR, TG, and CRP. Additionally, it exhibited a direct relationship with higher levels of total cholesterol, LDL-C, and HDL-C. Similar pattern were observed in regularly-cycling women, where AMH varied inversely with WC, fasting insulin, and HOMA-IR. Notably, these associations between AMH and cardio-metabolic indices were predominantly influenced by BMI in both PCOS and non-PCOS individuals [12]. In another study involving 87 women diagnosed with PCOS and 53 healthy control subjects, no significant relationship were found between AMH levels and obesity, indices of IR, or variables related to MetS in both groups [33]. The variations in findings may arise from differences in the studied populations, research methodologies, measurement techniques, statistical analyses employed, and lack of adjustment for potential confounders.

A limited number of studies have assessed the association between WC and AMH with inconclusive results. Some studies have reported an inverse association between these measures of adiposity and AMH [12, 34], while others have observed no significant association [29, 35, 36]. In the context of our study, where we examined changes in WC over time across different age-specific AMH tertiles, we found that although there was a rising trend in WC among all women, regardless of their AMH tertile, there were no significant changes in WC when comparing different age-specific AMH tertiles. These findings suggest that AMH levels may not directly influence alterations in WC. The inconsistent findings among various studies may be attributed to differences in sample characteristics, measurement techniques, or confounding factors that were not accounted for. Further research is required to gain a better understanding of the relationship between WC, AMH, and potential underlying factors that contribute to the conflicting results seen in various studies.

Evidence also yielded inconsistent and inconclusive results regarding the association between AMH and glycemic parameters [5, 7, 15]. Our study detected a notable connection between age-specific AMH levels and fluctuations in FPG among women. Specifically, we observed that women in the highest tertile of age-specific AMH exhibited greater average in FPG compared to those in the lowest tertile of age-specific AMH. Although the study initially enrolled women with regular and predictable menstrual cycles, it did not exclude those with subclinical forms of PCOS characterized by elevated serum levels of AMH. This inclusion of participants at risk for metabolic disturbances due to PCOS remains a notable aspect of the study. However, after considering potential confounding factors through adjustment, this association lost statistical significance, suggesting that the initial relationship was likely influenced by these variables. Additionally, our analysis revealed an interaction effect (Time × age-specific AMH tertile), indicating that the impact of age-specific AMH tertiles on FPG diminishes over time. Therefore, despite the initial observation of a significant relationship between age-specific AMH levels and FPG fluctuations, our findings suggest that this association may be confounded by other factors and is subject to change over time. These results underscore the complexity of the relationship between AMH and glycemic parameters, highlighting the need for further research to elucidate the underlying mechanisms and identify potential confounders that might influence these associations.

In this study, we explored how age-specific AMH levels influenced the lipid profile in women. We found that the mean TG levels did not differ significantly across age-specific AMH tertiles, even though they increased over time. However, intriguing findings emerged regarding the effects of age-specific AMH on HDL-C levels. Women in the second and third age-specific AMH tertiles exhibited lower mean HDL-C compared to those in the first tertile. This association remained significant only for the second tertile of age-specific AMH and the passage of time after adjusting for confounding factors. The possible mechanism underlying this association may involve the effects of AMH on ovarian function and steroidogenesis. AMH is a marker of ovarian reserve and reflects the number of antral follicles in the ovaries [37]. AMH also inhibits the aromatization of androgens to estrogens, which may affect the lipid profile and cardiovascular risk in women [38]. Estrogens have been shown to increase HDL-C levels and protect against atherosclerosis [39]. Therefore, women with higher age-specific AMH levels may have lower estrogen levels and lower HDL-C levels compared to women with lower age-specific AMH levels. This may explain why women in the second tertile of age-specific AMH had lower mean HDL-C than those in the first tertile, even after adjusting for confounding factors. The disappearance of the significant association between the third tertile of age-specific AMH and HDL-C after controlling for potential confounders may imply that other factors, such as age, genetic variation, or environmental exposure, may also affect the relationship between AMH and HDL-C [40, 41]. Further studies are needed to elucidate the mechanisms and implications of this association.

High BP is another common feature of MetS that increases the risk of CVD and T2D. The association between BP and AMH remains inconclusive, with some studies reporting a reverse significant association [7, 34], while others find no association [42, 43] or a positive association [44]. Our findings suggest that regardless of AMH, there were no significant differences in the mean changes of SBP and DBP over time among women in the second and third tertiles of age-specific AMH. Moreover, the interaction between time and age-specific AMH tertile on the mean changes of BP was not significant.

Based on our results, although there were a few minor variations observed in the Mets component concerning age-specific AMH levels, our study has revealed no significant correlation between serum levels of this hormone and the occurrence of MetS. This finding remained unchanged even after accounting for potential confounding variables. This means that variations in age-specific AMH levels do not appear to play a substantial role in the development of MetS.

To the best of our knowledge, this study represents the first community-based prospective investigation into the risk of MetS in relation to serum concentrations of AMH among women of reproductive age in a general population. The study possesses several notable strengths. Firstly, it adopts a longitudinal community-based design, ensuring a comprehensive understanding of the subject matter. Additionally, the study has a lengthy follow-up duration with several interval follow up visits, enabling the observation of long-term trends. Moreover, the researchers accounted for significant confounding factors. Another strength of our study is that we used age-specific values for AMH rather than crude AMH to assess the association with MetS and its components. This approach can account for the age-related decline of AMH and provide more accurate estimates of ovarian reserve and lipid metabolism in women and employed age-specific values of AMH [26]. These approaches facilitated the identification of longitudinal associations between variables while effectively adjusting for confounding factors. Nonetheless, it is crucial to acknowledge certain limitations inherent in our study that should be taken into account when interpreting the results. We did not assess AMH levels on a specific day of the menstrual cycle. However, it is important to note that serum AMH concentrations remain consistent throughout the menstrual cycle, making it a valuable marker of fertility compared to other indicators. Another possible drawback was that we only took into account a single measurement of AMH for each case in our group. Taking multiple measurements of AMH could be considered as an alternative that enhances the precision of determining the ovarian reserve status for each individual [6]. Additionally, our results may have been influenced by unmeasured genetic and lifestyle factors due to lack of available data on these variables. Future studies should strive to incorporate genetic and lifestyle information to further elucidate the relationship between AMH and MetS.

## Conclusions

Our study findings reveal that there were no significant differences in the hazard ratio of MetS among different age-specific AMH tertiles. Although we observed slight variations in the mean changes of specific MetS components, such as FPG and HDL-C, these differences did not exert a substantial impact on the overall HR of MetS. Consequently, further research is warranted to gain a more comprehensive understanding of the intricate association between AMH and metabolic health outcomes. By expanding our knowledge base in this area, future studies may help elucidate the clinical utility, if any, of incorporating AMH assessment into routine cardiometabolic risk screening protocols. Until then, healthcare professionals should continue to rely on well-established risk factors and evidence-based guidelines when evaluating and managing metabolic health in their patients.

## Data Availability

The datasets created during the present study can be obtained from the corresponding author upon making a reasonable request.

## Abbreviations

MetS: Metabolic syndrome
CVD: Cardiovascular disease
T2D: Type 2 diabetes
POI: Premature ovarian insufficiency
AMH: Anti-Müllerian hormone
FRS: Framingham Risk Score
PCE: Pooled Cohort Equations
PCOS: Polycystic ovary syndrome
TLGS: Tehran Lipid and Glucose Study
EIA: Enzyme immunoassay
FPG: Fasting plasma glucose
TG: Triglycerides
HDL-C: High-density lipoprotein cholesterol
CVs: Coefficients of variation
MAQ: Modified activity questionnaire
SD: Standard deviation
IQR: Interquartile range
HR: Hazard Ratio
WC: Waist circumference
SBP: Systolic blood pressure
DBP: Diastolic blood pressure
GEE: Generalized Estimating Equation models
HOMA-IR: Homeostasis model assessment of insulin resistance
